# High duty cycle moth sounds jam bat echolocation: bats counter with compensatory changes in buzz duration

**DOI:** 10.1242/jeb.244187

**Published:** 2022-09-22

**Authors:** Yohami Fernández, Nicolas J. Dowdy, William E. Conner

**Affiliations:** ^1^ Department of Biology, Wake Forest University, 1834 Wake Forest Road, Winston-Salem, NC 27109, USA; ^2^ Department of Zoology, Milwaukee Public Museum, 800 West Wells Street, Milwaukee, WI 53233, USA

**Keywords:** Jamming behavior, Biosonar, Duty cycle, *Eptesicus fuscus*, *Bertholdia trigona*, *Arctiinae*

## Abstract

Tiger moth species vary greatly in the number of clicks they produce and the resultant duty cycle. Signals with higher duty cycles are expected to more effectively interfere with bat sonar. However, little is known about the minimum duty cycle of tiger moth signals for sonar jamming. Is there a threshold that allows us to classify moths as acoustically aposematic versus sonar jammers based on their duty cycles? We performed playback experiments with three wild-caught adult male bats, *Eptesicus fuscus*. Bat attacks on tethered moths were challenged using acoustic signals of *Bertholdia trigona* with modified duty cycles ranging from 0 to 46%. We did not find evidence for a duty cycle threshold; rather, the ability to jam the bat's sonar was a continuous function of duty cycle consistent with a steady increase in the number of clicks arriving during a critical signal processing time window just prior to the arrival of an echo. The proportion of successful captures significantly decreased as the moth duty cycle increased. Our findings suggest that moths cannot be unambiguously classified as acoustically aposematic or sonar jammers based solely on duty cycle. Bats appear to compensate for sonar jamming by lengthening the duration of their terminal buzz and they are more successful in capturing moths when they do so. In contrast to previous findings for bats performing difficult spatial tasks, the number of sonar sound groups decreased in response to high duty cycles and did not affect capture success.

## Teaser

**Highlighted Article:** Video recordings and acoustic stimulation show that bat foraging success decreases in the presence of high duty cycle moth signals; bats alter their echolocation to compensate.

## INTRODUCTION

**Fig. 1.**
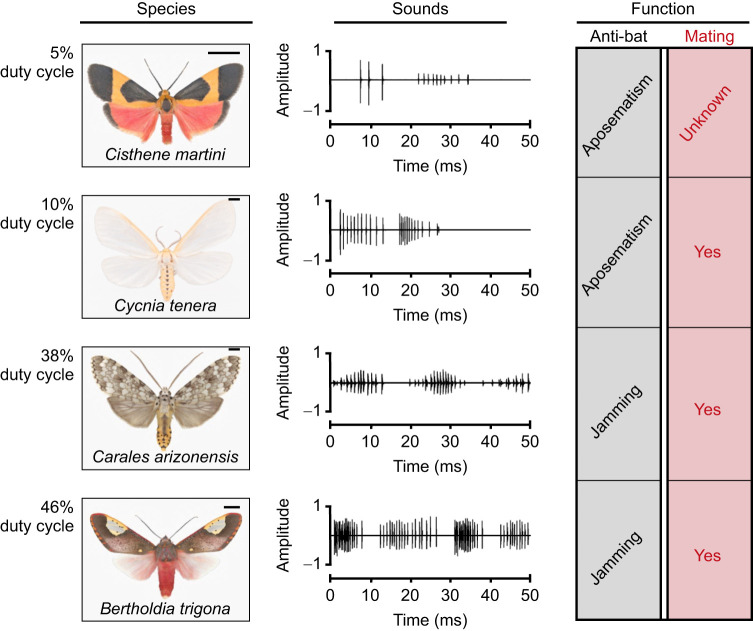
**Diversity in tiger moths acoustic signals.** Each row displays information from one species. Left column: species name, image and maximum duty cycle produced in their signals. Center column: oscillogram of the tymbal sounds. Right column: reported function of acoustic signals as anti-bat defense and the presence of acoustic mating. Maximum duty cycle values were obtained from Barber and Conner (2006) and Corcoran et al. (2010). The role of sound in both behavioral contexts was obtained from Conner (1999), Corcoran et al. (2010), Dowdy and Conner (2016) and Fernández et al. (2020). Scale bars: 3 mm.

Bats, with the combination of flight and an echolocation system, became the dominant aerial predators of nocturnal lepidopterans (Simmons and Stein, 1980; Jones and Holderied, 2007) over 65 million years ago (Conner and Corcoran, 2012). Their echolocation signals impose strong selective pressures on moths that have driven the evolution of ultrasound-sensitive ears, able to detect bat signals and trigger escape maneuvers (Roeder, 1962; Fullard, 1998; Kawahara et al., 2019). Subsequently, as a second line of defense against bats, tiger moths (Erebidae: Arctiinae) evolved sound-producing organs – tymbals – and the ability to produce broadband clicks in response to bat cries (Blest et al., 1963; Dunning and Roeder, 1965). Tymbal sounds are produced by modified cuticular thoracic plates, controlled by underlying muscles (Blest et al., 1963; Fullard and Heller, 1990). As the muscles contract and relax, the tymbal organs buckle inward and outward, respectively, producing two trains of clicks separated by a silent period. These sounds vary greatly between species (Fig. 1) in their frequency characteristics, the number of clicks produced during one tymbal activation cycle, their duty cycle (percentage of time occupied by sound in a 100 ms time window) and function (Barber and Conner, 2006; Corcoran et al., 2010; Dowdy and Conner, 2019), including their use in sexual acoustic communication (Conner, 1999; Nakano et al., 2013; Fernández et al., 2020). Based on previous findings, moth clicks were divided into low duty cycle signals (<20%) with few clicks per tymbal activation, and high duty cycle signals (>20%) with many clicks (Corcoran et al., 2010; Kawahara and Barber, 2015), suggesting that anti-bat moth sounds function in different ways. This variation in duty cycle is due to a combination of morphological differences in the tymbal organ and the rate of activation of this structure (Dowdy and Conner, 2019). Some species, for example, possess tymbals with a high number of striations, which results in the production of a high number of clicks per modulation cycle, while others have lost this striated pattern and produce only a couple of clicks. The effect of interspecific variation of duty cycle on bats' foraging success is the focus herein.

Early observations have suggested that bats can learn to associate moth clicks with their chemical defenses (Hristov and Conner, 2005a,b; Barber and Conner, 2007) and consequently these sounds serve an aposematic and/or mimetic function (Dowdy and Conner, 2016; Barber et al., 2022). Tymbal sounds can also jam bat sonar, protecting moths from predation (Corcoran et al., 2011; Corcoran and Conner, 2012). The efficacy of the jamming behavior requires moth clicks to arrive at the bat's ear within a short time window (2 ms) just prior to the arrival of the echo (Miller, 1991; Tougaard et al., 1998). Arctiine signals produced at high duty cycles would have a higher probability of falling inside the 2 ms critical jamming window and thus more effectively interfere with bat sonar. Corcoran et al. (2009) demonstrated the survival advantage of high duty cycle signals in *Bertholdia trigona* over silent controls. However, whether moth duty cycle is correlated with increased capture error remains unclear.

When performing difficult spatial tasks, such as capturing tethered insects close to background vegetation, bats can modify their echolocation strategy by increasing the duration of the buzz and the production of sonar sound groups (SSG) (Moss et al., 2006). In general, SSGs could be defined as clusters of two or more echolocation calls with a relatively constant pulse interval, surrounded by calls with larger intervals (Moss et al., 2006; Hiryu et al., 2010; Kothari et al., 2014) (see Fig. S1 for an example echolocation sequence with SSGs). Similar signal modifications have been described when bats attempt to resolve the location of moving versus stationary prey (Kothari et al., 2014; Hulgard and Ratcliffe, 2016). These findings suggest that the temporal control over their echolocation sequence could be necessary to deal with complex and dynamic situations.

Additionally, bats are constantly exposed to acoustic interference, which can be considered as a different type of complexity. To overcome acoustic interference, bats can also display flexibility in their echolocation strategies, which improves the signal-to-noise ratio and decreases the ambiguity between their calls and the jamming signals (Amichai et al., 2015; Jones et al., 2018). Bats not only change the frequency properties of their calls (similar to the jamming avoidance response of weakly electric fish; Bullock et al., 1972) but also emit longer and louder echolocation calls to deal with jamming in the form of a Lombard response (Ulanovsky et al., 2004; Amichai et al., 2015; Jones et al., 2018; Pedersen et al., 2022). These observations have been described under bat–bat jamming conditions, and we here examine the compensatory behaviors of bats to moth jamming for the first time.

Two hypotheses drove our research: (1) that there would be a threshold duty cycle necessary for effective sonar jamming and that this threshold would allow us to classify moths as sonar jammers based solely on their duty cycles (Corcoran et al., 2010; Conner and Corcoran, 2012), and (2) that bats would compensate for sonar jamming by (a) lengthening their terminal buzz and/or (b) adding SSGs as observed in response to other complex tasks (Moss et al., 2006; Hulgard and Ratcliffe, 2016).

We did not find evidence for a duty cycle threshold; rather, the ability to jam bat sonar appeared to be a continuous function of duty cycle, rejecting hypothesis 1. Big brown bats (*Eptesicus fuscus*) increased the length of their terminal buzz when faced with high duty cycles, supporting hypothesis 2a, but did not add SSGs, rejecting hypothesis 2b.

## MATERIALS AND METHODS

### Animals

Playback experiments were performed with three wild-caught adult male big brown bats, *Eptesicus fuscus* (Beauvois, 1796). Animals were captured in Forsyth County, NC, USA, under state collecting permit 16-SC01070. Bats were housed together in wooden cages, at Wake Forest University (WFU), in a temperature-controlled room (∼25°C) on a 12 h:12 h light:dark cycle. Individuals were fed mealworms (*Tenebrio molitor* larvae) and adult female greater wax moths (*Galleria mellonella*) nightly. Moths were acquired as larvae from King's Wholesale Bait (Liberty, IN, USA) and reared to adulthood. Live mealworms were obtained from The Nature's Way (Greenbay, WI, USA). After a 2 week period, the bats were released at their site of collection. The WFU Animal Care and Use Committee approved all procedures described for the behavioral experiments (A16-127).

### Playback experimental setup

Playback experiments took place in an outdoor flight arena (18×5.5×3 m L×W×H) on the WFU campus. *Galleria mellonella* females were deafened by ablating their tympanic membranes, so that escape behaviors in response to bat echolocation would not be enacted. The moths were tethered by the abdomen to a fine monofilament line (1 m) attached to the ceiling of the flight cage. Individual bats were trained to remove food items (mealworms and adult moths) from the tether prior to the initiation of playback experiments. Bat echolocation cries produced during attacks on the tethered female wax moths were recorded using an ultrasonic microphone connected to an Ultrasound Gate 416H (Avisoft Bioacoustics, Berlin, Germany), operated by a computer running Avisoft RECORDER USGH, sampling at 250 kHz (Fig. S2). The microphone was placed 1 m above the moth.

### Acoustic stimuli

**Fig. 2.**
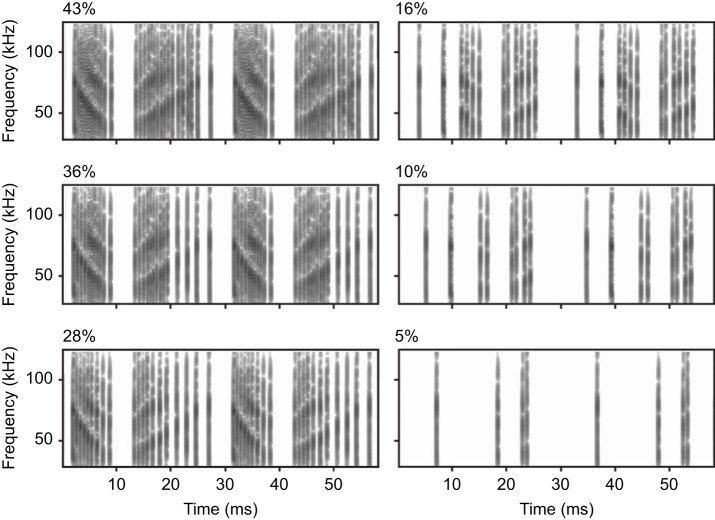
**Example spectrograms of the acoustic stimuli broadcast during playback experiments.** Each panel represents a modified signal obtained from previously recorded clicks of *Bertholdia trigona*. Signals were modified to generate moth clicks at different duty cycles (shown top right). Six of the 13 utilized stimuli are displayed. Spectrograms were obtained using a sampling rate of 250 kHz and a window length of the fast Fourier transform (FFT) of 256 points.

Acoustic stimuli were generated in Matlab R2015a (The MathWorks, Inc., Natick, MA, USA). Each stimulus was derived from a natural modulation cycle (MC) previously recorded from the jamming tiger moth, *B. trigona* (at a 250 kHz sample rate; N.J.D., unpublished) and altered using a custom-written Matlab script. This script was written to create modified MCs which varied in duty cycle from 0 to 46% (calculation assumes a 100 ms window, 0.3 ms click duration and 4 ms silent interval between successive modulation cycles) (Fig. 2). Other key acoustic characteristics defined in previous work such as sweep frequency, inter-cycle silent interval, and the active and passive half-modulation cycle durations were held constant (Corcoran et al., 2010). Duty cycle values were chosen to cover the range of ‘low’ (0–15%), ‘medium’ (16–30%) and ‘high’ (above 30%) duty cycles known to be produced by tiger moths (Barber and Conner, 2006; Corcoran et al., 2010; Dowdy and Conner, 2019).

For the playback experiments, stimulation files were randomly presented with an AT 100 ultrasonic speaker (Binary Acoustic Technology), located 1 m above the tethered moth. Ultrasonic clicks were broadcast with a root mean square (RMS) level of 80 dB sound pressure level (SPL), as measured with a ¼-inch Microtech Gefell microphone (model MK 301) at 1 m, connected to a conditioning measuring amplifier (Microtech Gefell MN-921). This amplitude is similar to those described for *B. trigona*'s sounds during an anti-bat response (Corcoran et al., 2010). The directionality of the speaker was measured during acoustic stimulation as a reference and a decrease of −20 dB SPL for a 20 deg angle was observed. Acoustic stimulation was triggered manually as the bat approached the target and the signal was played back continuously during the attack sequence. The timing of the stimulus initiation (during the search or approach phase of the echolocation sequence) was determined post-recording. The stimuli consisted of 15 categories, including silence as a control condition and 14 MCs at different duty cycles. Each stimulus category was presented at least 5 times for all bats combined.

### Video recording and behavioral scoring

Each bat–moth interaction was recorded with three calibrated high-speed, infrared-sensitive cameras (Basler Ace acA-2000-50gmNIR, Ahrensburg, Germany) (Fig. S2). Video was acquired with StreamPix6 software (Norpix, Inc., Montreal, QC, Canada) setup with a 6 s recording buffer, at 80 frames s^−1^ with 1280×720 pixel resolution. The recording buffer allowed us to record 3 s of activity before and after the TTL triggering event. Camera recordings were synchronized to each other and audio recording via a TTL pulse generated with custom hardware (Innovision Systems, Columbiaville, MI, USA). A fourth camera equipped with a telephoto lens was used to get a more detailed view of the interaction space, allowing us to clearly distinguish between attacks that failed as a result of abortion of the attack and those that failed because of miscapture (defined below). The flight arena was illuminated with three Raytec Raymax 200 platinum infrared illuminators (Ashington, UK).

Video recordings were reviewed, and the outcome was scored as (1) successful capture, (2) miscapture or (3) aborted attacks. An interaction was considered a successful capture when the bat captured the moth in the tail or wing membrane without releasing it. Miscaptures were differentiated from aborted attacks by the bat's flight behavior. During a miscapture, bats would extend their tail or wing membranes in anticipation of prey capture without contacting the moth. In aborted attacks, individuals would fail to enact the prey capture sequence described above or they would reduce their speed and/or change direction completely, avoiding contact with the target. Trials in which bats simply flew directly past targets, without showing any interest in the tether, were not scored. Although bats could perform multiple capture attempts, only the first interaction of every trial was analyzed.

### Audio analysis

The playback timing was reviewed in the audio recordings and only those files in which the acoustic stimulus was triggered prior to the terminal buzz were included in the analysis. Bat attack phases were defined based on the inter-pulse interval (IPI) of the echolocation calls as: search phase (IPI longer than 50 ms); approach phase (IPI between 49 and 13 ms) and terminal buzz phase (IPI shorter than 13 ms) (Corcoran et al., 2009; Jones et al., 2018). We selected the first capture attempt of every trial for acoustic analysis. The duration of the terminal buzz phase was determined from the beginning of the first pulse to the end of the last one.

The SSGs were identified during the approach phase of each echolocation sequence and the number of calls per SSG was determined. For each interaction, we calculated the average number of calls produced per SSG and this value was used for further statistical analysis. Following the criteria of Kothari et al. (2014), we defined SSGs as clusters of two or more calls with a relatively constant IPI between them (within 5% error with respect to the mean IPI of the sound group) that are flanked by longer IPI (at least 1.2 times the mean interval within the call cluster). In this case, only those audio files in which the acoustic stimulus was triggered at the end of the echolocation search phase and continued during the approach phase were used for the analysis. We also classified each emitted SSG into one of three categories depending on their number of calls: doublets, triplets and multi-call. Doublets and triplets contained two and three pulses, respectively, within one strobe. The multi-call category included those SSGs with four or more calls in it. The number of SSGs produced per category for every trial, in response to different acoustic stimulations, was measured.

All audio recordings were analyzed in Avisoft SASLab Pro v5.2. We used the Automatic Parameter tool to automatically identify the acoustic signals in the audio recordings. The echoes of individual calls, the second and third harmonic and the stimuli were manually removed to facilitate automatic detection.

### Statistical analysis

Statistical analyses of observation data were performed in R version 3.5.2 (http://www.R-project.org/). Means are reported with the standard deviation of the mean. Where *P*-values were adjusted, we opted for the conservative Bonferroni correction method when performing multiple comparisons. Adjusted *P*-values greater than 1 are reported as 1. The standard alpha of 0.05 was used. We interpreted our results within the gradual language of evidence outlined by Muff et al. (2022).

A generalized linear mixed model (GLMM), using the *glmer* function from the *lme4* package (http://www.R-project.org/), was utilized to create a model to assess the effect of duty cycle and duration of the terminal buzz phase on the percentage of successful capture attempts. The response variable (outcome of bat–moth interactions) was treated as a binary variable (possible outcomes: successful or failed capture). The independent variables (moth sound duty cycle and duration of terminal buzz phase) were treated as continuous variables. The predictor variable (buzz duration) was logarithmically transformed to improve model accuracy. Bat identity was also included as a random effect to account for the lack of independence in using individuals for multiple trials.

To detect whether successful capture rate was affected by the number of SSGs produced by the bats while foraging and the number of calls included in each SSG, we used a GLMM similar to the one described above. The outcome of the interactions was also used as the response variable, which was treated as a binary variable; and bat identity was included as a random variable.

Because the normality assumptions were not met, a Kruskal–Wallis test was performed to analyze the effect of moth signal duty cycle on the average number of SSGs produced by the bats, as well as on the number of calls emitted per SSG. Dunn's test was used for *post hoc* analysis in the case of a significant Kruskal–Wallis test. A linear regression was also generated to determine whether the acoustic stimulation with different duty cycles had a significant effect on the average number of doublets, triplets and multi-call SSGs produced.

## RESULTS

**Fig. 3.**
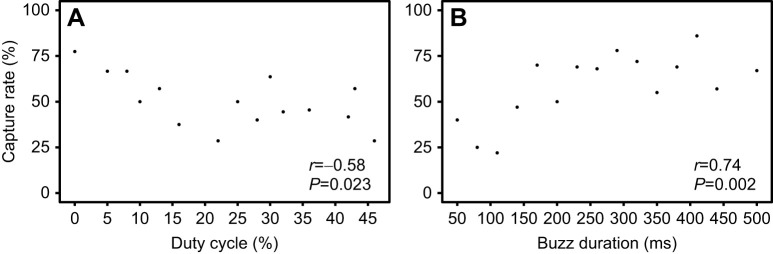
**Effect of moth duty cycle and bat terminal buzz duration on the successful capture rate of *Eptesicus fuscus* (*N*=3).** (A) Correlation of the successful capture rate and the duty cycle of *B. trigona* signals (Pearson's correlation: *r*_13_=−0.58, *P*=0.023). The percentage of successful captures decreased as duty cycle increased. (B) Correlation between the percentage of successful captures and the duration of the terminal buzz phase (Pearson's correlation: *r*_13_=−0.74, *P*=0.002). Bats were more successful at capturing targets when buzz duration increased.

We analyzed 247 bat–moth interactions from three individual male bats over nine alternating nights. To understand the effect of moth signal duty cycle on bat performance, we presented 14 simulated moth signals with different duty cycles during bat attacks on tethered moths and measured the successful capture rate for each condition. We also examined the acoustic responses of attacking bats during silent (control) conditions (133/247) and during playback stimulation with moth signals (114/247). The duration of the terminal buzz phase in the bat attack sequence was measured for each duty cycle value for both successful and unsuccessful captures. Logistic regression was used to predict bat performance based on moth signal duty cycle and bat buzz duration. We found very strong evidence for a negative effect of the duty cycle of moth signals on capture success (β=−0.040, *P*<0.001; Table 1). The proportion of successful captures decreased as the moth duty cycle increased (Fig. 3A). Bats showed the highest rate of successful captures (77%) for the control condition (0% duty cycle) and the odds of successful captures declined about 4% for each 1% increase in duty cycle (Table 1).

**Table 1.**
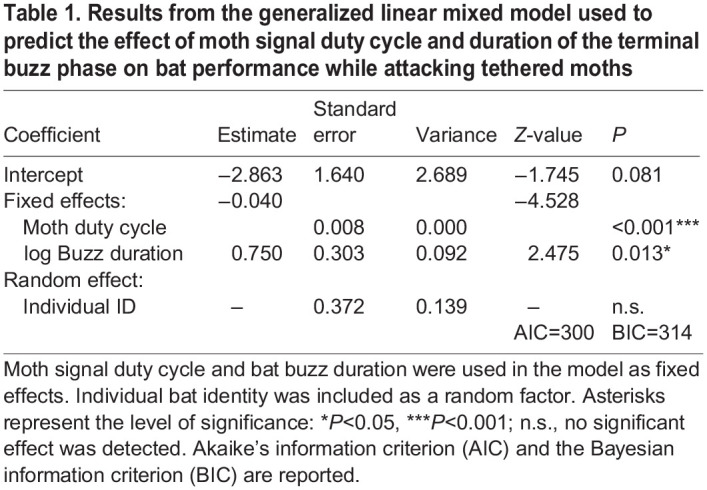
Results from the generalized linear mixed model used to predict the effect of moth signal duty cycle and duration of the terminal buzz phase on bat performance while attacking tethered moths

**Fig. 4.**
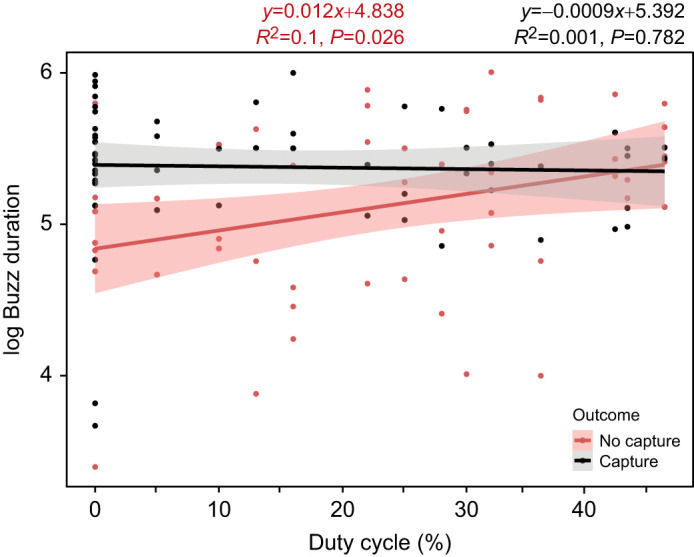
**Effect of moth duty cycle on the duration of the terminal buzz phase of *E. fuscus* (*N*=3).** The duration of this foraging phase (buzz duration, ms) increased as duty cycle increased for the unsuccessful (red) capture attempts. The linear relationships are shown with solid lines and the shaded areas represents the 95% confidence interval. Equations, *R*^2^ coefficients and *P*-values are reported.

Bat performance was also affected by buzz duration. We found moderate evidence for a positive relationship between the duration of the terminal buzz phase and successful capture rate (β=0.750, *P*=0.013; Table 1). Bats were more likely to be successful capturing tethered moths when they produced high buzz durations (Fig. 3B). Bats had the lowest percentage of captures for short buzz durations (between 50 and 110 ms) and their performance increased by 8% for each additional 100 ms of buzz duration. There was moderate evidence for buzz duration differences related to the duty cycle of the moth's signals when bats missed their targets (*F*_1,49_=5.286, *P*=0.026). In these situations, bats produced longer buzzes when prompted with high duty cycle moth signals (Fig. 4). During successful capture attempts, however, we observed that bats tended to produce long buzzes independently of the stimulus condition.

We analyzed 225 audio recordings from bat–moth interactions to detect the emission of SSGs (see Materials and Methods). In 90% of the analyzed files (202/225), SSGs were produced during the approach phase. The number of SSGs per echolocation sequence varied from 1 to 9 with an average of 2.7±2.0. We also examined bat foraging performance during attacks on tethered moths based on the production of SSGs and number of calls per SSG, but no significant effect was observed (number of SSGs: β=−0.022, *Z*=−0.269, *P*=0.787; number of calls per SSG: β=−0.021, *Z*=−0.173, *P*=0.862; AIC=311).

**Fig. 5.**
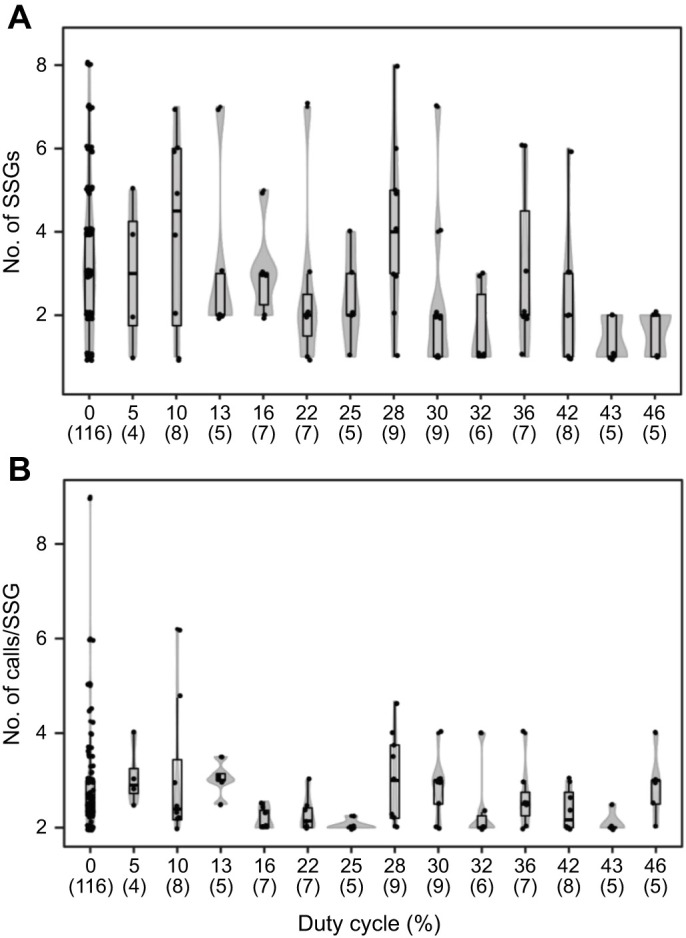
**Number of sonar sound groups (SSG) and calls per SSG produced during *E. fuscus* (*N*=3) attack sequences on tethered moths.** Violin plots of (A) the number of SSGs and (B) the number of calls per SSG produced during playback experiments in relation to the moth duty cycle presented for stimulation. Actual data from each experimental trial are displayed as points jittered along the midline of their respective box plot. The gray curve represents the kernel distribution of the data. Box plot upper and lower hinges represent the 25th and 75th percentiles of their respective distributions. The 50th percentile (median) is shown as a thicker black line between the hinges. Tukey-style whiskers extend from each hinge to the most extreme value within 1.5× the interquartile range (IQR). The number of replicates for each duty cycle is displayed within parentheses on the *x*-axis.

**Fig. 6.**
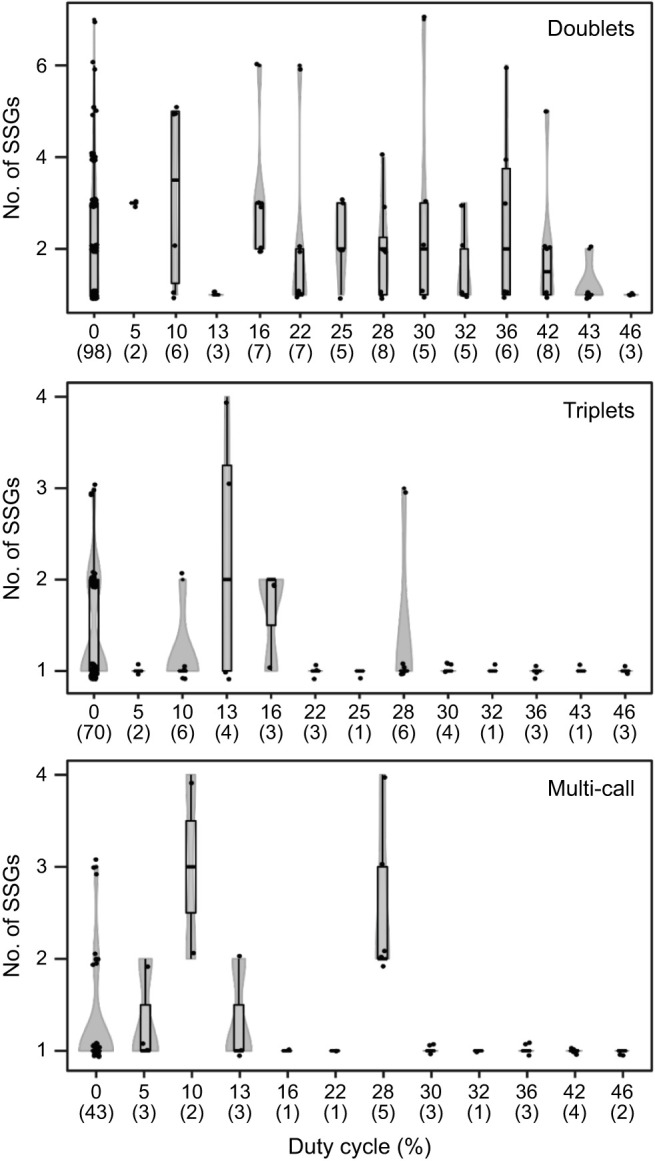
**Variability of the type of SSG produced by *E. fuscus* (*N*=3) in relation to the duty cycle of tiger moth signals.** Strobes were classified into three categories according to the number of calls within one strobe: doublets, triplets or multi-call. Each panel shows violin plots of the number of SSGs produced per category. Raw data are displayed as points jittered along the midline of their respective box plot. The number of replicates for each duty cycle is displayed within parentheses on the *x*-axis.

There was no evidence that duty cycle of moth signals had an effect on the mean number of SSGs produced by bats (Kruskal–Wallis test: χ^2^_13_=26.3; *P*=0.015; Dunn's test: *P*>0.05) (Fig. 5A). The number of calls per SSG was not affected by moth sound duty cycle either (Kruskal–Wallis test: χ^2^_13_=31.9; *P*=0.002; Dunn's test: *P*>0.05) (Fig. 5B). The average number of calls emitted per SSG was 2.4±1.2. For a better understanding of the number of calls produced per SSG in response to different acoustic simulations, we measured the total number of doublets, triplets and multi-call SSGs per trial. From a total of 616 analyzed SSGs, 61% were classified as doublets, 24% were triplets and 15% were multi-call (see Fig. S1 for SSG categories). A higher number of doublets was also observed when we analyzed the SSGs produced by each individual independently (Table 2). Although the total number of SSGs produced for each category (doublets, triplets and multi-call) tended to decrease when the bats were stimulated with high duty cycle signals (Fig. 6), we only observed a significant decrease for the triplets (*F*_1,200_=13.57, *P*<0.001).

**Table 2.**
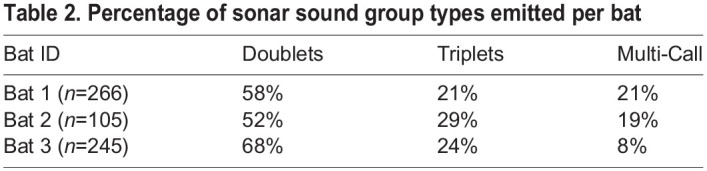
Percentage of sonar sound group types emitted per bat

## DISCUSSION

By broadcasting synthetic signals of *B. trigona* with different duty cycles to bats attacking tethered moths, we determined that higher duty cycle signals were more effective at diminishing foraging performance. We originally hypothesized that moth signals should exceed a duty cycle threshold to effectively jam bat sonar. This idea was supported by previous results showing that tiger moths could be separated into two clusters based on the duty cycle of their signals (Corcoran et al., 2010). The first group (tentatively labeled as moths producing aposematic sounds and their mimics) includes species producing clicks below 20% duty cycle, while the second group produces duty cycle signals above 20% and were considered the cluster of jamming species (see also Kawahara and Barber, 2015). However, our results do not support the existence of a duty cycle threshold that would, on its own, classify signals as either aposematic or jamming, rejecting our hypothesis 1. We found a concomitant decline in the successful capture of moths by bats as moth signal duty cycle increases.

Behavioral experiments with *E. fuscus* have demonstrated that clicks presented within 2 ms before the return of an echo affect the distance-discrimination ability of these bats (Miller, 1991; Tougaard et al., 2004). Tougaard and co-workers (1998) also found evidence of acoustic interference in the bat brain when arctiine clicks were presented in this time window. According to those studies, the arrival of a single click at the correct temporal position relative to the echo would be enough to cause acoustic interference (Miller, 1991; Tougaard et al., 1998, 2004). Emissions from *Cycnia tenera*, a species that falls within the low duty cycle cluster with a maximum value of 8.5% (Barber and Conner, 2006; Corcoran et al., 2010), were shown to interfere with bat echolocation and result in target-range miscalculations by free-flying bats (Ratcliffe and Fullard, 2005). These findings, in concert with our current observations, suggest that the classification of species as sonar jammers solely based on their maximum duty cycles is subjective. Nonetheless, signals with higher duty cycles are more likely to arrive within the critical time window. Aposematic clicks like those produced by *C. tenera* can serve a jamming function as Ratcliffe and Fullard (2005) proposed, but their effectiveness will likely be weaker than that for species which produce sound at higher duty cycles. All sound-producing moths are capable of disrupting sonar to some extent, but some signals (i.e. species) are more effective than others and this is, at least in part, driven by increasing duty cycle. Going forward, our results suggest that sonar jamming should be interpreted within a probabilistic framework rather than as a binary trait.

Extrapolation of these conclusions should take into consideration the bat sample size of the current study. Only three individual bats were included in the playback experiment and their behavioral response patterns were similar among individuals. Although it is not unusual in bat studies to have a small sample size, it can be a form of sampling bias. Future studies involving a larger number of bats as well as bat species would provide further evidence about the relationship between moth duty cycle and bat capture performance. Another factor that might influence our results is the position of the speaker, 1 m away from the tethered moth. This type of stimulation setup has previously been used successfully in tests of bat–bat interspecific jamming behavior (Jones et al., 2018). With this experimental setup, bats could potentially avoid the effect of the stimuli by attacking the moth from above, thereby performing spatial release from masking. This behavior, however, was not frequently observed in the current study. Bats approached the target from above in only 5% of the total interactions, and in some cases they did it during the control trials where no sound was emitted, suggesting that this maneuver was unrelated to the emission of the moth sounds. In most of the interactions, the animals opted for a horizontal approach to their target.

While bat foraging performance was negatively affected by duty cycle, we also gathered evidence that the duration of the buzz phase significantly affected the outcome of bat–moth interactions, which supports hypothesis 2a. Big brown bats do appear to compensate for sonar jamming by lengthening the duration of their terminal buzz. When producing longer buzzes, bats were more successful at capturing moths. These observations are consistent with previous studies showing an increase in the duration of the echolocation pulses as a jamming avoidance response in bat–bat interactions. Amichai and co-workers (2015) demonstrated that bats shifted their echolocation signals towards longer durations and higher intensities when exposed to jamming conditions by conspecific individuals. This acoustic modification would result in a better signal-to-noise ratio and it also facilitates target detection (Kalko and Schnitzler, 1993; Amichai et al., 2015). Pulse duration increases have also been described in *E. fuscus* as a response to heterospecific jamming behavior (Jones et al., 2018). Our study focused on the duration of the entire terminal buzz phase; however, this is directly related to the duration of each of their pulses, the IPI and the total number of pulses produced. We also found that unsuccessful bats tended to increase their buzz duration as they were stimulated with tiger moth signals of increasing duty cycle (see Fig. 4). This was particularly apparent for moth duty cycles below 20–25%. Successful bats capture moth targets by shifting their buzz duration towards higher values for all duty cycles presented. In some cases, lengthening the buzz duration may not be totally effective (see overlap of linear regression lines in Fig. 4) to overcome the jamming effect of moth sounds.

Changes in buzz duration have been also reported when bats perform tasks of different complexity. In *Myotis daubentoni*, total buzz duration, as well as buzz I and buzz II subphases, were longer for bats tracking moving airborne prey than for those hunting stationary prey in the air or in the water (Hulgard and Ratcliffe, 2016). The authors suggest that buzz duration might reflect the degree of difficulty of a given foraging task. Similar results were observed in *E. fuscus*, which uses shorter buzzes to catch still, tethered prey (Moss et al., 2006). Task complexity could also be defined in relation to the foraging background and its level of clutter. Buzz duration during target approach has been observed to decrease within more cluttered backgrounds (Moss et al., 2006; Hulgard and Ratcliffe, 2014). Instead, bats resolve this complex task by increasing the production of SSGs during the approach phase (Moss et al., 2006; Kothari et al., 2014). The use of SSGs gives the bat more rapidly updated information under conditions where this is needed. By modulating their IPI, bats create a depth of field (Petrites et al., 2009). The pulses produced within the strobe at short IPIs bring information from objects close to the bat, while the long IPIs flanking the SSG are used to test farther surroundings and plan their future course (Petrites et al., 2009).

Surprisingly, we found that the number of SSGs did not increase while attempting what could be construed as a complex task (i.e. attacking tethered moths when stimulated with high duty cycle playback). However, a similar trend was seen when bats were flying inside corridors of varying width (Wheeler et al., 2016; Accomando et al., 2018). The complexity of the task was set by adjusting the corridor width and the number of obstacles the bats had to maneuver. The proportion of SSGs produced with two pulses (doublets) decreased as the corridor width became narrower (Wheeler et al., 2016; Accomando et al., 2018). Because most of the SSGs produced were doublets, this can be interpreted as a decrease in the total percentage of strobes used by the bats as task complexity increased. In our scenario, the bats were tasked with processing their own echoes in addition to the train of simulated moth clicks, increasing the complexity of foraging. Perhaps decreasing the number of SSGs could be a strategy to decrease the amount of acoustic stimulation perceived by the bat. This suggests that different types of task complexity (clutter versus target-produced jamming signals) may require different solutions on the part of the echolocating bat.

## Supplementary Material

10.1242/jexbio.244187_sup1 Supplementary information
