# Supplementary material for: High duty cycle moth sounds jam bat echolocation: bats counter with compensatory changes in buzz duration
Source: J Exp Biol. 2022 Sep 22;225(18):jeb244187. doi: 10.1242/jeb.244187 (PMC9637272; doi:10.1242/jeb.244187)
Supplement: Supplementary information [file jexbio-225-244187-s1.pdf]

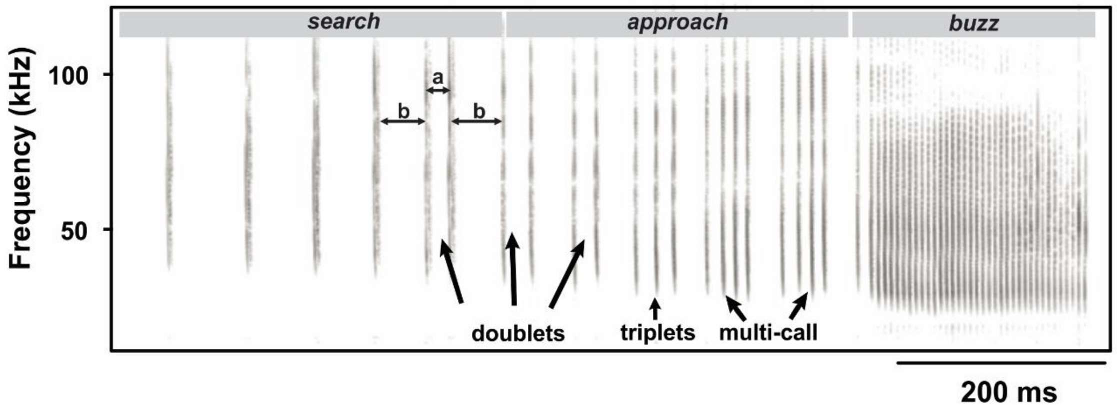

Fig. S1. Echolocation sequence of *Eptesicus fuscus* during an attack on a tethered moth. Representative calls from each foraging phase are shown (i.e. search, approach and terminal buzz phases). Six sonar strobe groups were produced in this sequence: three doublets, one triplet and two multi-calls (see black arrows). Doublets and triplets consisted of pairs and groups of three pulses respectively. The multi-call category included sonar strobe groups with four or more pulses. In this example, both multi-calls included four pulses. Letters (a and b) represent an example of the difference in the inter-pulse intervals that characterized SSGs. An SSG was defined as a group of two or more calls separated by a short inter-pulse interval (a) and flanked by longer inter-pulse intervals (b). The spectrogram was obtained with a sampling rate of 250 kHz and a window length of the Fast Fourier Transform (FFT) of 1024 points.

Journal of Experimental Biology • Supplementary information

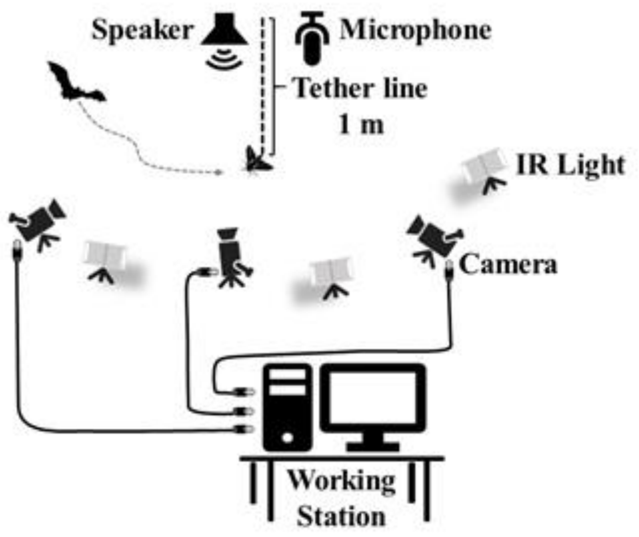

Fig. S2. Diagram of the recording setup in the outdoor flight arena during playback experiments.
